# Ocular surface health in Shanghai University students: a cross-sectional study

**DOI:** 10.1186/s12886-018-0825-z

**Published:** 2018-09-12

**Authors:** Shanshan Li, Jiangnan He, Qiuying Chen, Jianfeng Zhu, Haidong Zou, Xun Xu

**Affiliations:** 1 Shanghai Eye Disease Prevention and Treatment Center, Shanghai, China; 2 Shanghai General Hospital, Shanghai, China

**Keywords:** Ocular surface health, Eye health, Dry eye disease, Anxiety, Students

## Abstract

**Background:**

Our study aimed to investigate the ocular surface health of Shanghai University students.

**Methods:**

This is a cross-sectional study carried out among freshmen and sophomores on the main campus of Shanghai University. Questionnaires including the widely-used ocular surface disease index (OSDI) and the Zung Self-rating Anxiety Scale (SAS) were completed first, and then ocular examinations were conducted regarding height & weight, blood pressure and heart rate, optometry, intraocular pressure exam, vision and subjective refraction, Aladdin, Macular pigment density measurement, tear test, anterior segment examination, fundus photography, ophthalmologist check, TOPCON OCT check, and Collin’s fundus blood test.

**Results:**

Totally 901 students were involved in our five-day study. The prevalence of myopia was 92% (the spherical equivalent refraction (SER) < − 0.50 D), and that of high myopia was 23% (SER < − 6.0D). The prevalence of dry eye disease (DED) was 10%. The corneal epithelial loss rate (corneal fluorescein staining > 1) was 10%, and corneal sensation decline rate (≤ 30 mm) was 12%. 4.5% of subjects (*n* = 40) had moderate or severe anxiety, 78% were mild and a small portion (17.5%) didn’t have anxiety at all. No statistical significant association was found between anxiety with DED, fluorescein staining or with corneal sensation (all *p* > 0.05). However, subjects with DED had more symptoms of anxiety. Results also showed that students who kept eye strain for a long time were more inclined to have DED (12.5%: 6.9%, *p* = 0.0407, 95% CI); those who watched mobile phones and/or computers for over eight hours daily were more vulnerable to DED and fluorescein staining than others (14.1%: 8.6%, *p* = 0.0129; 13.0%: 8.3%, *p* = 0.0233, 95% CI).

**Conclusions:**

Keeping eye strain or near work for a long time is associated with DED, while students with DED tend to encounter anxiety symptoms. The prevalence of myopia in Chinese university students is still high. We consider it necessary to provide education to university students about the good eye-using habits, and to diagnose anxiety for student patients with DED.

**Electronic supplementary material:**

The online version of this article (10.1186/s12886-018-0825-z) contains supplementary material, which is available to authorized users.

## Background

Eye health, including ocular surface health, is an integral part of the human physical health. Statistics from the World Health Organization (WHO) alarms us against the severity of eye health. A total of nearly 50 million persons are blind and another 150 million are victims of severe visual disability, and this number will probably double in the year 2020 [1].

China, with its 1.5 billion population, has a huge number of students. As reported in the 2016 National Statistical Communiqué on Development of Education published by the Ministry of Education, there were about 160 million students from grade 1 to 12, and over 41 million college/university students (including nearly 2 million post-graduate students) at school at the survey time [2]. College students form a distinctive group with their own obvious characteristics. They are young in relatively good physical conditions (having passed the physical examination for entering the colleges); have finished 12-year basic education with good academic scores; have more opportunities (compared with elementary and middle school students) and spare time to use video display terminals (VDT) at a short distance for a long time; often stay up late; and many of them bear the pressure of studying. As revealed by the General Administration of Sport in the Communiqué on National Physical Health Monitoring conducted in 2014, physical fitness of college students in China is worse than the year 2010, while the prevalence of myopia was higher with a tendency to younger ages at the time of incidence [3].

According to the 2004 National Students Physical Health Monitoring Report, the poor sight detection rate was 32.5% among pupils aged 7 to 12. The rate was 59.4% among junior middle school students and 77.3% among senior middle school students. A larger proportion (80.0%) of university students were detected with poor sight and some of them even had high myopia [4]. High myopia often causes pathological changes of eyes with symptoms like decline of eye sight, fast worsening of myopia, proptosis, bad dark adaptation, dark shadows, etc. Complications may include macular degeneration, retinal detachment and other diseases that may cause blindness [5, 6].

With the popularity of computers and smart phones/video display terminals (VDT), more and more Chinese students suffer from refractive errors and have to wear glasses if the error is not detected and corrected in a timely manner [7]. While wearing glasses is an obvious sign of damaged eye health, some ocular surface diseases (OSD) are more concealed, as well as fundus oculi diseases.

Dry eye is a multifactorial disease characterized by unstable tear film causing a variety of symptoms and/or visual impairment, potentially accompanied by ocular surface damage. In 2016, the Asia Cornea Society (ADES) and the Dry Eye Society Japan implemented new diagnostic criteria for DED that enabled diagnosis with two positive items, namely subjective symptoms and decreased TBUT (≤ 5 s) [8].

To date, teenagers-based studies in China have been focusing on myopia/high myopia, good examples of which may be the Shunyi Study [9, 10], the Guangzhou Study [11], and the Yangxi Study [12]. On the other hand, college/ university-based studies are rare, though Jing Sun et al. had carried out a survey on diopter and myopia in Shanghai Donghua University in 2012 [13]. Our study aimed to investigate the ocular surface health of Shanghai University students.

The obvious strength of the updated study is that it is the first to pay attention to ocular surface health condition of Chinese university students, as well as to explore the association of their anxiety status with their eye health.

## Methods

This is an observational cross-sectional study, comprising a questionnaire survey and ophthalmologic examinations. It was carried out on Baoshan Campus of Shanghai University in September 2016. Located in Shanghai with three campuses, Shanghai University is one of the national key universities with over 54,000 students from various areas across the country to study in the university by February 27, 2017 [14]. Baoshan Campus is the main and largest campus containing almost all the colleges/departments where all their freshmen and sophomores receive undergraduate education. Our study is designed to include only freshmen and sophomores rather than senior students, because we had a 3-year visit plan for students with eye diseases. Students of higher grades would graduate and leave the school pretty soon, making the 3-year visit plan difficult to carry out. The school, the students’ union and the Youth League Committee were responsible for informing the students, who visited our booths on the campus at their own willingness. Subjects first read and signed the informed consent, then completed a questionnaire on iPad designed and provided by the investigators. Incomplete questionnaires could not be submitted. After completing the questionnaires (see Additional file 1) including but not limited to the widely-used ocular surface disease index (OSDI) and the Zung Self-rating Anxiety Scale (SAS) [15], subjects went through examinations, i.e. height & weight, optometry, intraocular pressure exam, vision and subjective refraction (< 0.8, one eye), Aladdin (Topcon, made in Japan), Macular pigment density measurement, tear test, anterior segment examination, fundus photography, ophthalmologist check, TOPCON (made in Japan, TOPCON CORPOERATION) OCT check, Collin’s fundus blood test, blood pressure and heart rate. Around ten medical staff worked together with five doctors to implement the study on site. Examination data were logged into computer by a data entry operator. The survey lasted for five days from September 9 through September 13, 2016.

Statistical analysis was performed with SAS® software, version 9.2 (SAS Institute, NC, USA). Spherical equivalent refraction (SER) was calculated as the spherical value of the refractive error plus half of the cylindrical value. Myopia was defined as a SER ≤ 0.5 diopters (D) and high myopia was defined as a SER ≤ 6.0 D. The OSD was determined based on the OSDI scores and the ophthalmologic examination results. Corneal epithelial loss was identified when the corneal fluorescein staining > 1. Corneal sensation ≤ 30 mm was considered as decline. Statistical analysis focused on OSDs, specifically the dry eye disease (DED), cornea fluorescein staining and corneal sensation. All *P* values were two-sided and were considered statistically significant when the P values were < 0.05. Person Chi-Square test was used to analyze associations with genders and ages, different eye-using habits and anxiety status. Cochran-Armitage Trend Test was performed to identify the trend of association between anxiety and DED.

## Results

Our study involved 901 students in total in Shanghai University. The subjects, at an equal sex ratio, responded with questionnaires and ophthalmologic examinations. Most of the subjects were freshmen and sophomores whose ages ranged from 18 to 22 (Table 1), and no one was under the age of 17, as per the automatic calculation of the system after dates of birth were entered.

**Table 1:** Demographics and eye examination results by disease types and in total

		Dry eye disease			Cornea fluorescein staining			Corneal sensation		
	Total (*N* = 901)	Absent (*N* = 733)	Present (*N* = 94)	*P*-value	≤ 1 (*N* = 796)	>1 (*N* = 89)	*P*-value	>30 mm (*N* = 780)	≤ 30 mm (*N* = 105)	*P*-value
Age, years	19.7 (2.7)	19.7 (2.7)	19.9 (2.5)	0.4721^a^	19.7 (2.7)	19.8 (2.5)	0.5520^a^	19.7 (2.7)	19.7 (2.5)	0.8036^a^
Age, years [n (%)]				0.6914^b^			0.0860^b^			0.8003^b^
< 18	102 (11.3%)	85 (11.6%)	6 (6.4%)		88 (11.1%)	14 (15.7%)		91 (11.7%)	11 (10.5%)	
18 ≤ age < 19	322 (35.7%)	264 (36.0%)	34 (36.2%)		294 (36.9%)	21 (23.6%)		275 (35.3%)	40 (38.1%)	
19 ≤ age < 20	124 (13.8%)	101 (13.8%)	13 (13.8%)		107 (13.4%)	14 (15.7%)		109 (14.0%)	12 (11.4%)	
20 ≤ age < 21	94 (10.4%)	77 (10.5%)	11 (11.7%)		85 (10.7%)	8 (9.0%)		85 (10.9%)	8 (7.6%)	
21 ≤ age < 22	89 (9.9%)	70 (9.5%)	12 (12.8%)		73 (9.2%)	14 (15.7%)		76 (9.7%)	11 (10.5%)	
≥ 22	170 (18.9%)	136 (18.6%)	18 (19.1%)		149 (18.7%)	18 (20.2%)		144 (18.5%)	23 (21.9%)	
Gender [n (%)]				0.4143^b^			0.6014^b^			0.7361^b^
Male	397 (44.1%)	321 (43.8%)	37 (39.4%)		354 (44.5%)	37 (41.6%)		343 (44.0%)	48 (45.7%)	
Female	504 (55.9%)	412 (56.2%)	57 (60.6%)		442 (55.5%)	52 (58.4%)		437 (56.0%)	57 (54.3%)	
Height(cm)	158.3 (37.9)	159.0 (36.9)	157.2 (38.1)	0.6505^a^	158.4 (37.7)	157.7 (39.3)	0.8593^a^	159.3 (35.8)	151.7 (50.0)	0.0560^a^
Weight(Kg)	54.9 (16.6)	55.2 (16.6)	53.9 (15.3)	0.4793^a^	55.0 (16.3)	55.0 (19.0)	0.9694^a^	55.4 (16.1)	52.2 (19.8)	0.0649^a^
BMI(Kg/m^2)	19.7 (5.2)	19.7 (5.2)	19.6 (4.9)	0.8058^a^	19.7 (5.1)	19.6 (5.9)	0.9738^a^	19.8 (5.0)	18.6 (6.4)	0.0216^a^
Axial length R, mm	25.0 (2.6)	25.0 (2.7)	25.0 (2.8)	0.9968^a^	25.0 (2.6)	24.9 (2.9)	0.7163^a^	25.1 (2.3)	24.6 (4.4)	0.1407^a^
Axial length L, mm	24.8 (3.0)	24.8 (3.0)	24.9 (2.8)	0.8640^a^	24.8 (2.9)	24.6 (3.9)	0.5279^a^	24.8 (2.8)	24.5 (4.4)	0.3616^a^
Intraocular pressure R, mmHg	13.9 (3.4)	13.9 (3.4)	13.7 (3.4)	0.5859^a^	13.9 (3.4)	14.0 (3.2)	0.7750^a^	13.9 (3.3)	13.7 (3.8)	0.5143^a^
Intraocular pressure L, mmHg	14.0 (3.5)	14.1 (3.4)	13.5 (3.4)	0.1393^a^	14.0 (3.5)	13.8 (3.3)	0.6580^a^	14.0 (3.5)	13.8 (3.4)	0.6178^a^
Diopter R, D	398.7 (259.5)	400.3 (256.3)	408.2 (257.9)	0.7782^a^	400.4 (264.8)	377.4 (205.1)	0.4278^a^	397.7 (259.4)	400.7 (260.9)	0.9119^a^
Diopter L, D	368.4 (264.7)	365.8 (265.1)	404.0 (237.4)	0.1835^a^	368.4 (269.8)	361.7 (215.6)	0.8189^a^	366.4 (261.8)	377.7 (286.8)	0.6810^a^

*BMI* body mass index, *L* left eye, *R* right eye, *SD* standard deviation

^a^ANOVA

^b^Pearson Chi-Square test

The prevalence of myopia was 92% (the spherical equivalent refraction (SER) < − 0.50 D), and that of high myopia was 23% (SER < − 6.0D). The prevalence of DED was 10%. The corneal epithelial loss rate (corneal fluorescein staining > 1) was 10%, and corneal sensation decline rate (≤ 30 mm) was 12%.

Statistical analysis indicated that 4.5% of subjects (*n* = 40, see Table 3) had moderate or severe anxiety, 78% were mild and a small portion (17.5%) didn’t have anxiety at all. No statistical significant association was found between anxiety with DED, fluorescein staining or corneal sensation (all *p* > 0.05). However, subjects with DED had more symptoms of anxiety (see Table 3), such as feeling afraid for no reason at all (*p* = 0.0023), getting upset easily or feeling panicky (*p* = 0.0004), and being bothered by headaches neck and back pain (*p* = 0.0101), feeling weak and getting tired easily (*p* = 0.0004), being bothered by dizzy spells (p = 0.0004), having fainting spells or feeling like it (*p* = 0.0175), being bothered by a stomach aches or indigestion (*p* = 0.0001), face getting hot and blushes (*p* = 0.0039). Cochran-Armitage Trend Test identified a trend of anxiety among DED subjects (see Table 4). Adjusted *p*-values also showed worse anxiety status of subjects with DED, mainly with symptoms of getting upset easily or feeling panicky (*p* = 0.0216), feeling weak and getting tired easily (*p* = 0.0340), being bothered by dizzy spells (*p* = 0.0120), and being bothered by a stomach aches or indigestion (*p* = 0. 0190).

Results also showed that students who kept eye strain for a long time were more inclined to have DED (12.5%: 6.9%, *p* = 0.0407, 95% CI); those who watched mobile phones and/or computers for over eight hours daily were more vulnerable to DED and fluorescein staining than others (14.1%: 8.6%, *p* = 0.0129; 13.0%: 8.3%, *p* = 0.0233, 95% CI). No significant differences were found among other variables such as age, gender, near work and improper reading gestures; no significant differences/associations for corneal sensation (see Table 2). However, none of the comparison results in Table 2 was significant (*p* < 0.05) after adjustment.

**Table 2:** Prevalence of eye diseases by demographics, eye care habits and in total

	Dry eye disease %(95% CI)^a^ (*N* = 94)	*P*-value	Cornea fluorescein staining >1%(95% CI)^a^ (*N* = 89)	*P*-value	Corneal perception test ≤ 30 mm %(95% CI)^a^ (*N* = 105)	*P*-value
Age, years [n(%)]		0.6914^b^		0.0860^b^		0.8003^b^
< 18	6.6%(2.5,13.8)		13.7%(7.7,22.0)		10.8%(5.5,18.5)	
18 ≤ age < 19	11.4%(8.0,15.6)		6.8%(4.3,10.2)		12.7%(9.3,16.9)	
19 ≤ age < 20	11.4%(6.2,18.7)		11.3%(6.3,18.2)		9.7%(5.1,16.3)	
20 ≤ age < 21	12.5%(6.4,21.3)		8.5%(3.7,16.1)		8.5%(3.7,16.1)	
21 ≤ age < 22	14.6%(7.8,24.2)		15.7%(8.9,25.0)		13.5%(7.2,22.4)	
≥ 22	11.7%(7.1,17.8)		10.6%(6.4,16.2)		13.5%(8.8,19.6)	
Gender [n (%)]		0.4143^b^		0.6014^b^		0.7361^b^
Male	10.3%(7.4,14.0)		9.6%(6.9,12.9)		12.6%(9.5,16.3)	
Female	12.2%(9.3,15.5)		10.3%(7.8,13.3)		11.3%(8.7,14.4)	
Near work [n (%)]		0.1296^b^		0.8785^b^		0.9049^b^
No	10.0%(6.9,13.8)		10.6%(7.5,14.4)		11.2%(8.1,15.1)	
Yes	13.6%(10.5,17.2)		10.3%(7.7,13.4)		10.9%(8.2,14.2)	
View for long time [n (%)]		0.0683^b^		0.4039^b^		0.0169^b^
No	8.1%(4.5,13.2)		12.1%(7.7,17.7)		15.9%(10.9,22.1)	
Yes	13.2%(10.6,16.3)		9.9%(7.7,12.6)		9.6%(7.4,12.2)	
Incorrect reading gesture [n (%)]		0.0624^b^		0.5986^b^		0.2197^b^
No	9.5%(6.5,13.2)		9.8%(6.8,13.4)		9.5%(6.6,13.1)	
Yes	14.0%(10.8,17.6)		10.9%(8.2,14.1)		12.2%(9.4,15.6)	
Play computers or video games for a long time [n (%)]		0.7056^b^		0.5224^b^		0.6531^b^
No	11.6%(8.7,15.2)		11.1%(8.3,14.4)		10.6%(7.9,13.9)	
Yes	12.5%(9.2,16.5)		9.7%(6.8,13.2)		11.6%(8.5,15.4)	
Study for over 8 h every day [n (%)]		0.0303^b^		0.7818^b^		0.3854^b^
No	9.1%(6.5,12.2)		10.3%(7.7,13.5)		11.0%(8.3,14.2)	
Yes	13.9%(10.6,17.6)		9.8%(7.1,13.0)		12.9%(9.8,16.4)	
Use mobile phone/computer for over 8 h every day [n (%)]		0.0099^b^		0.0233^b^		0.6436^b^
No	9.1%(6.8,12.0)		8.3%(6.1,10.9)		12.3%(9.6,15.3)	
Yes	15.0%(11.2,19.5)		13.0%(9.6,17.1)		11.2%(8.0,15.1)	
Often stay up late [n (%)]		0.2960^b^		0.6277^b^		0.0517^b^
No	9.0%(5.0,14.6)		9.0%(5.1,14.5)		16.3%(11.0,22.8)	
Yes	11.9%(9.6,14.6)		10.3%(8.2,12.7)		10.8%(8.7,13.4)	
Often wear eye makeup [n (%)]		0.0541^b^		0.2297^b^		0.4444^b^
No	10.9%(8.8,13.3)		9.8%(7.9,12.0)		11.7%(9.6,14.0)	
Yes	21.1%(9.6,37.3)		15.8%(6.0,31.3)		15.8%(6.0,31.3)	
Do outdoor exercises, eye exercises or overlooking with eyes [n (%)]		0.3515^b^		0.7346^b^		0.6941^b^
No	12.9%(8.9,17.8)		10.5%(7.1,15.0)		12.5%(8.7,17.2)	
Yes	10.7%(8.3,13.4)		9.8%(7.6,12.4)		11.6%(9.2,14.3)	
Do you wear contact lenses? [n (%)]		0.3279^b^		0.0766^b^		0.7296^b^
No	11.5%(8.9,14.5)		9.2%(6.9,11.9)		11.5%(9.0,14.5)	
Yes	14.1%(9.6,19.8)		13.6%(9.2,19.2)		10.6%(6.7,15.8)	

*BMI* body mass index, *L* left eye. *R* right eye, *SD* standard deviation

^a^The Exact Unconditional Confidence Interval

^b^Pearson Chi-Square test

## Discussion

We carried out a cross-sectional study using both questionnaires and ophthalmologic examinations for 901 students in Shanghai University for the purpose of getting real data of ocular surface health of university students in China. The obvious strength of the updated study is that it is the first to pay attention to ocular surface health condition of Chinese university students, as well as to explore the association of their anxiety status with their eye health.

The study results showed that the prevalence of myopia was 92% (SER < − 0.50 D), that of high myopia was 23% (SER < − 6.0D), and dry eye disease 10%. The corneal epithelial loss rate (corneal fluorescein staining > 1) was 10%, and the corneal sensation decline rate (≤ 30 mm) was 12%.

There are quite a few published studies on myopia and ocular surface health of Chinese university students. The study implemented by Jing Sun et al. in Shanghai Donghua University found that 95.5% of the subjects were myopic (SER < − 0.50 D), 19.5% were highly myopic (SER < − 6.0 D) [13]. This is similar to our study results. There are no published study results on OSD conditions of Chinese university/college students yet. However, studies in other countries may provide references. A cross-sectional survey conducted in students from the University of Monterrey using Ocular Surface Disease Index (OSDI) questionnaire in 2016 have found that University students have a prevalence of 70.4% of ocular surface disease, and OSD was associated with gender (women have a higher prevalence), smoking and the use of eye drops [16]. Another study by Kofi Asiedu et al. concluded that the prevalence of symptomatic dry eye among undergraduate students in Ghana is high (44.3% (95% confidence interval [CI], 40.6–48.2%) and it is associated with self-medication with over-the-counter eye drops, allergies, use of oral contraceptive, windy conditions, very low humid areas, air-conditioned rooms, and sex [17]. Compared to those two studies, prevalence of OSDs in our study was not high. It may be because we took into consideration the ophthalmologic examination results to determine the OSD, instead of using OSDI scores only. We had investigated the contact lens wearing of these students, and analysis was reported in Table 2. However, no significant relations were found between contact lens wearing and DED, cornea fluorescein staining or corneal perception (*P* > 0.05), which may be due to the shorter and less frequent use of contact lenses by these freshmen.

Mental disorder, specifically anxiety, was explored in our study. As currently known, these factors are related to anxiety: Biological factors, such as genetic and physiological diseases, social factors, tension, and high work pressure, psychological factors, hormone levels, and so on. Published results of studies have already found correlation of eye diseases, especially the DED, with mental disorders. A study by Li M et al. in 2011 showed that the prevalence of anxiety or depression symptoms in dry eye syndrome subjects was significantly higher than in the control group (*p* = 0.003; *p* < 0.001, respectively) [18]. A meta-analysis concluded that depression and anxiety are more prevalent in DED patients than in controls, irrespective of the underlying etiologies of DED and ethnic differences of the patients [19]. Investigators of our study have also noticed, from their clinical practice, that many patients with ocular surface diseases had mental disorders to some extent. However, no significant association was found with DED, fluorescein staining or corneal sensation (all *p* > 0.05). A reasonable explanation may be that the subjects included in our study were young people (mean age 19.7 ± 2.7) with relatively good physical and mental health (they passed the university entrance examination and relevant physical checks). Results of a study by van der Vaart et al. also found associations between DED and anxiety differed across age groups with the elders having stronger associations [20].

Nonetheless, subjects with DED in our study were found to have more symptoms of anxiety, e.g. feeling afraid for no reason at all, getting upset easily or feeling panicky, feeling weak and getting tired easily, etc. (see Table 3). A statistically significant trend was found between anxiety and DED (see Table 4). This is in agreement with clinical experience. Patients with DED usually have more complaints and discomforts than those who only have refractive error. In addition to artificial tears and other medication, it is necessary for doctors to explain reasons and console them. For student patients, eye irritation symptoms such as foreign body sensation and dryness are common as a result of over-regulation of the eyes and/or reduction of blinks [21] during/after excessive reading or computer/mobile phone use. Some students may grow anxious as these symptoms occur from time to time.

**Table 3:** Various mental statuses by disease types and in total

		Dzry eye disease			Cornea fluorescein staining			Corneal sensation		
	Total (*N* = 901)	Absent (*N* = 733)	Present (*N* = 94)	*P*-value	≤ 1 (*N* = 796)	>1 (*N* = 89)	*P*-value	>30 mm (*N* = 780)	≤ 30 mm (*N* = 105)	*P*-value
I feel more nervous and anxious than usual.				0.1332^a^			0.9415^a^			0.4303^a^
A little or some of the time	491 (56.1%)	414 (57.1%)	46 (48.9%)		442 (56.1%)	49 (55.7%)		437 (56.5%)	54 (52.4%)	
Good part or most of the time	385 (43.9%)	311 (42.9%)	48 (51.1%)		346 (43.9%)	39 (44.3%)		336 (43.5%)	49 (47.6%)	
I feel afraid for no reason at all.				0.0023^a^			0.6147^a^			0.6648^a^
A little or some of the time	684 (78.4%)	576 (79.8%)	62 (66.0%)		614 (78.1%)	70 (80.5%)		605 (78.6%)	79 (76.7%)	
Good part or most of the time	189 (21.6%)	146 (20.2%)	32 (34.0%)		172 (21.9%)	17 (19.5%)		165 (21.4%)	24 (23.3%)	
I get upset easily or feel panicky.				0.0004^a^			0.7419^a^			0.5343^a^
A little or some of the time	551 (63.0%)	472 (65.0%)	43 (46.2%)		497 (63.2%)	54 (61.4%)		489 (63.3%)	62 (60.2%)	
Good part or most of the time	324 (37.0%)	254 (35.0%)	50 (53.8%)		290 (36.8%)	34 (38.6%)		283 (36.7%)	41 (39.8%)	
I feel like I’m falling apart and going to pieces.				0.1458^a^			0.5045^a^			0.7820^a^
A little or some of the time	757 (86.3%)	634 (87.3%)	77 (81.9%)		679 (86.1%)	78 (88.6%)		669 (86.4%)	88 (85.4%)	
Good part or most of the time	120 (13.7%)	92 (12.7%)	17 (18.1%)		110 (13.9%)	10 (11.4%)		105 (13.6%)	15 (14.6%)	
I feel that everything is all right and nothing bad will happen.				0.3091^a^			0.1519^a^			0.4895^a^
A little or some of the time	142 (16.2%)	122 (16.9%)	12 (12.8%)		123 (15.6%)	19 (21.6%)		128 (16.6%)	14 (13.9%)	
Good part or most of the time	732 (83.8%)	600 (83.1%)	82 (87.2%)		663 (84.4%)	69 (78.4%)		645 (83.4%)	87 (86.1%)	
My arms and legs shake and tremble.				0.3031^a^			0.3375^a^			0.5465^a^
A little or some of the time	761 (87.2%)	633 (87.8%)	79 (84.0%)		688 (87.5%)	73 (83.9%)		674 (87.4%)	87 (85.3%)	
Good part or most of the time	112 (12.8%)	88 (12.2%)	15 (16.0%)		98 (12.5%)	14 (16.1%)		97 (12.6%)	15 (14.7%)	
I am bothered by headaches neck and back pain.				0.0101^a^			0.2039^a^			0.6391^a^
A little or some of the time	503 (57.5%)	432 (59.7%)	43 (45.7%)		458 (58.2%)	45 (51.1%)		446 (57.8%)	57 (55.3%)	
Good part or most of the time	372 (42.5%)	292 (40.3%)	51 (54.3%)		329 (41.8%)	43 (48.9%)		326 (42.2%)	46 (44.7%)	
I feel weak and get tired easily.				0.0004^a^			0.6428^a^			0.1817^a^
A little or some of the time	322 (36.8%)	281 (38.8%)	19 (20.2%)		292 (37.0%)	30 (34.5%)		278 (36.0%)	44 (42.7%)	
Good part or most of the time	554 (63.2%)	444 (61.2%)	75 (79.8%)		497 (63.0%)	57 (65.5%)		495 (64.0%)	59 (57.3%)	
I feel calm and can sit still easily.				0.3764^a^			0.2911^a^			0.7117^a^
A little or some of the time	274 (31.4%)	232 (32.0%)	25 (27.5%)		251 (32.0%)	23 (26.4%)		240 (31.2%)	34 (33.0%)	
Good part or most of the time	598 (68.6%)	492 (68.0%)	66 (72.5%)		534 (68.0%)	64 (73.6%)		529 (68.8%)	69 (67.0%)	
I can feel my heart beating fast.				0.0550^a^			0.9174^a^			0.6683^a^
A little or some of the time	653 (74.5%)	545 (75.2%)	62 (66.0%)		587 (74.5%)	66 (75.0%)		578 (74.8%)	75 (72.8%)	
Good part or most of the time	223 (25.5%)	180 (24.8%)	32 (34.0%)		201 (25.5%)	22 (25.0%)		195 (25.2%)	28 (27.2%)	
I am bothered by dizzy spells.				0.0004^a^			0.8430^a^			0.5927^a^
A little or some of the time	692 (78.7%)	587 (80.7%)	61 (64.9%)		622 (78.6%)	70 (79.5%)		613 (79.0%)	79 (76.7%)	
Good part or most of the time	187 (21.3%)	140 (19.3%)	33 (35.1%)		169 (21.4%)	18 (20.5%)		163 (21.0%)	24 (23.3%)	
I have fainting spells or feel like it.				0.0175^a^			0.8478^a^			0.2952^a^
A little or some of the time	772 (88.1%)	646 (89.1%)	75 (80.6%)		695 (88.2%)	77 (87.5%)		678 (87.7%)	94 (91.3%)	
Good part or most of the time	104 (11.9%)	79 (10.9%)	18 (19.4%)		93 (11.8%)	11 (12.5%)		95 (12.3%)	9 (8.7%)	
I can breathe in and out easily.				0.8312^a^			0.4371^a^			0.9660^a^
A little or some of the time	424 (48.3%)	352 (48.5%)	44 (47.3%)		378 (47.9%)	46 (52.3%)		374 (48.3%)	50 (48.5%)	
Good part or most of the time	453 (51.7%)	374 (51.5%)	49 (52.7%)		411 (52.1%)	42 (47.7%)		400 (51.7%)	53 (51.5%)	
I get numbness and tingling in my fingers and toes.				0.9498^a^			0.8044^a^			0.5168^a^
A little or some of the time	763 (87.2%)	634 (87.3%)	81 (87.1%)		687 (87.3%)	76 (86.4%)		672 (86.9%)	91 (89.2%)	
Good part or most of the time	112 (12.8%)	92 (12.7%)	12 (12.9%)		100 (12.7%)	12 (13.6%)		101 (13.1%)	11 (10.8%)	
I am bothered by stomach aches or indigestion.				0.0001^a^			0.1914^a^			0.0392^a^
A little or some of the time	574 (65.4%)	494 (68.0%)	45 (47.9%)		522 (66.1%)	52 (59.1%)		498 (64.2%)	76 (74.5%)	
Good part or most of the time	304 (34.6%)	232 (32.0%)	49 (52.1%)		268 (33.9%)	36 (40.9%)		278 (35.8%)	26 (25.5%)	
I have to empty my bladder often.				0.3573^a^			0.0958^a^			0.8032^a^
A little or some of the time	609 (69.5%)	511 (70.6%)	62 (66.0%)		541 (68.7%)	68 (77.3%)		537 (69.4%)	72 (70.6%)	
Good part or most of the time	267 (30.5%)	213 (29.4%)	32 (34.0%)		247 (31.3%)	20 (22.7%)		237 (30.6%)	30 (29.4%)	
My hands are usually dry and warm.				0.6917^a^			0.2221^a^			0.4782^a^
A little or some of the time	288 (32.8%)	240 (33.1%)	33 (35.1%)		254 (32.2%)	34 (38.6%)		251 (32.4%)	37 (35.9%)	
Good part or most of the time	589 (67.2%)	486 (66.9%)	61 (64.9%)		535 (67.8%)	54 (61.4%)		523 (67.6%)	66 (64.1%)	
My face gets hot and blushes.				0.0039^a^			0.0704^a^			0.0303^a^
A little or some of the time	625 (71.3%)	528 (72.8%)	55 (58.5%)		555 (70.3%)	70 (79.5%)		543 (70.1%)	82 (80.4%)	
Good part or most of the time	252 (28.7%)	197 (27.2%)	39 (41.5%)		234 (29.7%)	18 (20.5%)		232 (29.9%)	20 (19.6%)	
I fall asleep easily and get a good night’s rest.				0.9241^a^			0.3199^a^			0.8477^a^
A little or some of the time	291 (33.1%)	243 (33.5%)	31 (33.0%)		266 (33.7%)	25 (28.4%)		256 (33.0%)	35 (34.0%)	
Good part or most of the time	587 (66.9%)	483 (66.5%)	63 (67.0%)		524 (66.3%)	63 (71.6%)		519 (67.0%)	68 (66.0%)	
I have nightmares.				0.3115^a^			0.2875^a^			0.2757^a^
A little or some of the time	637 (72.5%)	531 (73.0%)	64 (68.1%)		569 (71.9%)	68 (77.3%)		567 (73.1%)	70 (68.0%)	
Good part or most of the time	242 (27.5%)	196 (27.0%)	30 (31.9%)		222 (28.1%)	20 (22.7%)		209 (26.9%)	33 (32.0%)	

^a^Pearson Chi-Square test

**Table 4:** Summary of Zung self-rating anxiety scale

	Dry eye disease			Cornea fluorescein staining			Corneal sensation		
	Absent (*N* = 733)	Present (*N* = 94)	*P*-value	≤ 1 (*N* = 796)	>1 (*N* = 89)	*P*-value	>30 mm (*N* = 780)	≤ 30 mm (*N* = 105)	*P*-value
Severity of anxiety			0.0136^a^			0.1364^a^			0.8399^a^
None (<35)	85 (12.2%)	5 (5.6%)		85 (11.2%)	12 (14.3%)		88 (11.8%)	9 (9.3%)	
Mild (≥ 35,<55)	582 (83.4%)	76 (85.4%)		632 (83.6%)	71 (84.5%)		618 (83.2%)	85 (87.6%)	
Moderate (≥ 55,<65)	28 (4.0%)	6 (6.7%)		35 (4.6%)	0		33 (4.4%)	2 (2.1%)	
Severe (≥ 65)	3 (0.4%)	2 (2.2%)		4 (0.5%)	1 (1.2%)		4 (0.5%)	1 (1.0%)	
Missing	35	5		40	5		37	8	
Sum of scores			< 0.0001^b^			0.9229^b^			0.7769^b^
Number	698	89		756	84		743	97	
Mean (SD)	42.5 (7.2)	45.8 (7.5)		42.8 (7.3)	42.8 (7.0)		42.9 (7.4)	42.6 (6.6)	
Median	42.5	45.0		42.5	43.8		43.8	42.5	
Min: Max	25: 79	29: 66		25: 79	26: 65		25: 79	25: 65	

^a^CMH SCORES = RANK

^b^ANOVA

Therefore, school doctors, acting as the primary care taker for university students, are suggested to pay attention to mental statuses of students with DED symptoms rather than just cure DED alone. Consolation or soothing, or even psychological counseling can be performed whenever necessary. Medical-psychological health intervention mode may be adopted to provide science education, eye health survey, counseling and other interventions. To our knowledge, ours is the first study to call on school doctors to address anxiety status for university students with DED symptoms.

Regarding habits, statistical significance was found in two habits out of ten (see Table 2): (1) keeping eye strain for a long time, associated with the DED (*p* = 0.0407); and (2) watching mobile phones and/or computers for over 8 h every day, associated with the DED and corneal fluorescein staining (*p* = 0.0129, *p* = 0.0233). Near work, though considered as one of the reasons for refraction error [22–25], was found uncorrelated with DED, fluorescein staining or corneal sensation (*p* > 0.05). One possible explanation may be that the variable of near work is a vague expression without a specific standard for subjects to make better or precise judgments. Another reason may be near work itself doesn’t do much harm to ocular surface disease, but it will do if combined with a long time and/or continuous use of VDT, a good example of which is the variable “watching mobile phones and/or computers for over 8 hours every day”. Our study identified the importance of period of time, either for eye strain or watching VDTs. This is consistent with findings of previous studies [22, 25]. The blinking action prevents the lipid layer from contacting the mucus layer, maintains the thickness of the tear layer in the tear film, and keeps the tear film stable [26]. The number of blinks is reduced when eyes focus on close things. This is typical in VDT users. They tend to blink less when focus on computers and such terminals as a smart phone for a long time at a very short distance. This will cause an abnormal distribution of tears and tear secretion, which in turn leads to an increase in eye discomfort. Hence, it’s suggested to pay more attention to the duration of one continuous near work (watching or reading). Keeping time of the continuous near work as short as possible may be beneficial to students’ eye health. However, further studies may be needed to explore the exact length of time proper for one continuous near work.

Our study has obvious limitations. Firstly, the subjects were a specific group, and results may not be proper for a general population-based generalization. Besides, the sample size was not very big, which might cause statistical bias. Thirdly, only one university participated in the study. Future studies may involve more universities to cover as many majors as possible.

## Conclusions

Our study has revealed the fact the prevalence of myopia in Chinese university students is still high, that keeping eye strain or near work for a long time is associated with DED, and that students with dry eye disease/syndrome tend to encounter anxiety symptoms of one kind or another. This conclusion might be used by school doctors to educate university students about the good eye-using habits, and to diagnose anxiety for patients with DED. Further studies are warranted to cover more universities.

## Additional file

Additional file 1: Survey. College Students’ Eye Health Questionnaires including the widely-used ocular surface disease index (OSDI) and the Zung Self-rating Anxiety Scale (SAS). (DOCX 19 kb)

## Abbreviations

CI: Confidence interval
DED: Dry eye disease
OSD: Ocular surface diseases
OSDI: Ocular surface disease index
SAS: The Zung Self-rating anxiety scale
SER: Spherical equivalent refraction
VDT: Video display terminals
WHO: The World Health Organization
