# Supplementary material for: Ocular surface health in Shanghai University students: a cross-sectional study
Source: BMC Ophthalmol. 2018 Sep 12;18:245. doi: 10.1186/s12886-018-0825-z (PMC6134707; doi:10.1186/s12886-018-0825-z)
Supplement: Supplementary file 1 — Survey. College Students’ Eye Health Questionnaires including the widely-used ocular surface disease index (OSDI) and the Zung Self-rating Anxiety Scale (SAS). (DOCX 19 kb) [file 12886_2018_825_MOESM1_ESM.docx]

College Students' Eye Health Questionnaire

Basic Information

ID number:

Your name:

Your sex:

Your age:

PART 1 History of systemic disease and eye disease

A1. Do you have a history of chronic diseases?

1 No; 2 Hypertension; 3 Diabetes; 4 Cardiovascular and Cerebral Diseases

A2. Are there other myopia patients in your family?

1 Yes; 2 None (please skip to Part 2)

A3.1. Myopia in your family is (multiple choices)

1 Father; 2 Mother; 3 Grandpa (grandfather); 4 Grandma (grandmother)

Please fill in the diopter:

1 Father; 2 Mother; 3 Grandpa (Grandfather); 4 Grandma (Grandmother)

A3.2. Does anyone in your family have the following eye problems? If yes, please indicate the relationship with you.

1 no; 2 glaucoma; 3 cataracts; 4 high myopia; 5 other(please note)

Part II Vision Status and Eye Care

B1. How is your current vision status?

1 Myopia 2 Hyperopia 3 Astigmatism 4 Normal vision (if you choose 234, please go to Question 6)

B1.1. When did you find yourself nearsighted? (Please specify the specific grade after selection)

1 Kindergarten; 2 Elementary School, Grade ; 3 Junior High School, Grade ; 4 High School, Grade ; 5 University, Grade

B2. What do you think caused myopia? (Multiple choice)

1 genetic factors; 2 reading distance is too close; 3 too long time of reading; 4 incorrect reading posture; 5 long-term use of computers or game consoles

B3. Do you know what is the biggest harm of myopia?

1 see things blurred; 2 need to wear glasses, do not look good; 3 can not participate in intense sports activities; 4 will affect employment; 5 may lead to blindness; 6 other hazards; 7 I do not know

B4. Which of the following methods do you currently use to correct vision? (Multiple choices)

1 Frame Glasses; 2 Soft Contact Lenses; 3 Excimer Laser Surgery; 4 RGP (Hard Contact Lens); 5 OK Lens (Corner Plastic Lens); If you don’t choice 1, go directly to Question B5)

B4.1. Where did you get the first optometry and wear glasses?

1 hospital; 2 optical shops

B4.2. When was your first glasses?

1 Within 1 month after myopia occurred; 2 Nearly 6 months after myopia appeared; 3 More than 6 months after myopia occurred

B4.3. Where is the most recent optician?

1 Hospital; 2 Optical Shops; 3 Others

B4.4. If you are wearing frame glasses, have you considered the following vision correction methods?

1 Soft contact lens; 2 Excimer laser surgery; 3RGP and OK mirror; 4none

B5. Have you ever worn contact lenses?

1 No (If you have never worn a contact lens, please go to Question 6); 2 Yes

B5.1. What type of contact lens do you often wear?

1 day toss; 2 monthly toss; 3 seasons throw or above; 4 coloring tablets (US pupil); 5 others

B5.2. How often do you wear contact lenses?

1 everyday wear; 2 average 3 times per week; 3 weekly; 4 occasional

B5.3. Before wearing contact lenses, did you have eye examinations and post-hoc reviews in hospitals?

1 yes; 2 No

B5.4. Do you have dry eyes, foreign body sensation, red eyes and other symptoms when wearing coloring contact lenses or other contact lenses?

1 No; 2 Occasionally; 3 often

B5.5. When you feel uncomfortable, you will

1 Go to the hospital; 2 Buy eyedrops in pharmacy nearby; 3 Do not go to the hospital, do not use eye drops, just suspend contact lenses, wait until the symptoms disappear and wear again

B5.6. Do you wear contact lenses overnight?

1 never; 2 occasional; 3 often

B5.7. Do you have ever wear contact lenses longer than the limited life of the lenses?

1 never; 2 occasional; 3 often

B5.8. When you wear a contact lens, do you clean it properly in accordance with the standard operation of the contact lens?

1 Never; 2 Occasionally; 3 Often; 4 Always

B6. Do you ever feel eye discomfort in the last six months?

1 never; 2 occasional; 3 often (If you choose 1, please go to question B7)

B6.1. What are your eye discomfort symptoms? (Multiple choice)

1 Visual fatigue; 2 Eyes acid; 3 eyes bulge; 4 eye itch; 5 eye congestion; 6 photophobia; 7 tears; 8 dry eyes; 9 blurred vision; 10 other _______

B6.2. What choices do you take when you feel eye discomfort?

1 Go to a medical institution 2 Buy eye drops on your own 3 Do nothing

B7. Do you currently have the following habits?

1 study or work for more than 8 hours a day; 2 Use a mobile computer for a total of 8 hours or more; 3 Often sleep after 11pm; 4 Frequently eye makeup; 5 None

B8. Will you do some outdoor sports, eye exercises, visions, etc. after learning or playing computer games for a while?

1 Never; 2 Occasionally; 3 Often

B9. Are you satisfied with the current eye condition?

1 often; 2 occasional; 3 no; 4 not sure

Part III Eye Health Knowledge

C1. Do you pay attention to eye health information?

1 When the magazine, the network appears relevant information will read carefully; 2 When the eyes have problems, I will take the initiative to understand through books, networks, etc; 3 do not care

C2. What eye health informations are you interested in?

1 Myopia; 2 Myopia prevention; 3 Excimer laser surgery; 4 Glasses; 5 ; visual fatigue; 6 Other ________

C3. Does your school provide visual inspection?

1 Yes 2 No (If you choose 2, please go to Question C4)

C3.1. How often does the eye vision check provided by your school?

1 once per year; 2 once every 2 years; 3 once every 3 years; 4 none

C4. Have you received eye health education from the school?

1 Yes 2 No (If you choose 2, please go to Question C5)

C4.1. What is the eye health education provided by your school?

1 Consultation; 2 Clinic for free; 3 Related eye health information; 4 none

C5. Are you willing to undergo regular eye exams?

1 Yes 2 No

C6. How often do you think is appropriate to have regular eye examinations?

1 half year; 2 1 year; 3more than1 years

C7. Where do you prefer to receive eye health services?

1 School Hospital; 2 Ophthalmology Specialist Hospital; 3 General Hospital; 4 Professional Institutions Provide Special Services at School

C8. If school or hospital provides vision check and optician services, are you willing to participate?

1 Yes 2 No

C9. What do you think is the best way for your eye care health education?

1Read eye health books and information; 2Video or multimedia; 3Study through the Internet; 4Seminar held by the school

C10. What is your biggest concern for excimer laser surgery?

1 Safety; 2 Correct vision; 3 Postoperative recurrence; 4 Postoperative side effects; 5 Other __

Part IV OSDI Questionnaire

Have you experienced any of the of the of the of the of the of the following during the last week for question D1 to D5?

D1. Eyes that are sensitive to light?

4 All the time; 3 Most of the time; 2 Half of the time; 1 Some of the time; 0 None of the time

D2. Eyes that feel gritty?

4 All the time; 3 Most of the time; 2 Half of the time; 1 Some of the time; 0 None of the time

D3. Painful or sore eyes?

4 All the time; 3 Most of the time; 2 Half of the time; 1 Some of the time; 0 None of the time

D4. Blurred vision?

4 All the time; 3 Most of the time; 2 Half of the time; 1 Some of the time; 0 None of the time

D5. Poor vision?

4 All the time; 3 Most of the time; 2 Half of the time; 1 Some of the time; 0 None of the time

Have problems with your eyes limited you in performing any of the following during the last week for question D6 to D9?

D6. Reading?

4 All the time; 3 Most of the time; 2 Half of the time; 1 Some of the time; 0 None of the time

D7. Driving at night?

4 All the time; 3 Most of the time; 2 Half of the time; 1 Some of the time; 0 None of the time

D8. Working with a computer or bank machine (ATM)?

4 All the time; 3 Most of the time; 2 Half of the time; 1 Some of the time; 0 None of the time

D9. Watching TV?

4 All the time; 3 Most of the time; 2 Half of the time; 1 Some of the time; 0 None of the time

Have your eyes felt uncomfortable in any of the following situations during the last week for question D10 to D12?

D10. Windy conditions?

4 All the time; 3 Most of the time; 2 Half of the time; 1 Some of the time; 0 None of the time

D11. Places or areas with low humidity (very dry)

4 All the time; 3 Most of the time; 2 Half of the time; 1 Some of the time; 0 None of the time

D12. Areas that are air conditioned?

4 All the time; 3 Most of the time; 2 Half of the time; 1 Some of the time; 0 None of the time

Part V: Mental Health Status Survey ( Zung Self-Rating Anxiety Scale)

EI. feel more nervous and anxious than usual.

1 A little of the time; 2 Some of the time; 3 Good part of the time; 4 Most of the time

E2. I feel afraid for no reason at all.

1 A little of the time; 2 Some of the time; 3 Good part of the time; 4 Most of the time

E3. I get upset easily or feel panicky.

1 A little of the time; 2 Some of the time; 3 Good part of the time; 4 Most of the time

E4. I feel like I’m falling apart and going to pieces.

1 A little of the time; 2 Some of the time; 3 Good part of the time; 4 Most of the time

E5. I feel that everything is all right and nothing bad will happen.

1 A little of the time; 2 Some of the time; 3 Good part of the time; 4 Most of the time

E6. My arms and legs shake and tremble.

1 A little of the time; 2 Some of the time; 3 Good part of the time; 4 Most of the time

E7. I am bothered by headaches neck and back pain.

1 A little of the time; 2 Some of the time; 3 Good part of the time; 4 Most of the time

E8. I feel weak and get tired easily.

1 A little of the time; 2 Some of the time; 3 Good part of the time; 4 Most of the time

E9. I feel calm and can sit still easily.

1 A little of the time; 2 Some of the time; 3 Good part of the time; 4 Most of the time

E10. I can feel my heart beating fast.

1 A little of the time; 2 Some of the time; 3 Good part of the time; 4 Most of the time

E11. I am bothered by dizzy spells.

1 A little of the time; 2 Some of the time; 3 Good part of the time; 4 Most of the time

E12. I have fainting spells or feel like it.

1 A little of the time; 2 Some of the time; 3 Good part of the time; 4 Most of the time

E13. I can breathe in and out easily.

1 A little of the time; 2 Some of the time; 3 Good part of the time; 4 Most of the time

E14. I get numbness and tingling in my fingers and toes.

1 A little of the time; 2 Some of the time; 3 Good part of the time; 4 Most of the time

E15. I am bothered by stomach aches or indigestion.

1 A little of the time; 2 Some of the time; 3 Good part of the time; 4 Most of the time

E16. I have to empty my bladder often.

1 A little of the time; 2 Some of the time; 3 Good part of the time; 4 Most of the time

E17. My hands are usually dry and warm.

1 A little of the time; 2 Some of the time; 3 Good part of the time; 4 Most of the time

E18. My face gets hot and blushes.

1 A little of the time; 2 Some of the time; 3 Good part of the time; 4 Most of the time

E19. I fall asleep easily and get a good night’s rest.

1 A little of the time; 2 Some of the time; 3 Good part of the time; 4 Most of the time

E20. I have nightmares.

1 A little of the time; 2 Some of the time; 3 Good part of the time; 4 Most of the time
